# Influence of entrepreneurship support programs on nascent entrepreneurial intention among university students in China

**DOI:** 10.3389/fpsyg.2022.955591

**Published:** 2022-07-22

**Authors:** Shiyan Liao, Hasnain Javed, Lixin Sun, Muzaffar Abbas

**Affiliations:** ^1^School of Education, Zhaoqing University, Zhaoqing, China; ^2^Lahore College for Women University, Lahore, Pakistan; ^3^The Higher Education Research Office of Guangdong Academy of Education, Guangzhou, China; ^4^Department of Business Administration, Community College, Prince Sattam Bin Abdulaziz University, Al-Kharj, Saudi Arabia

**Keywords:** entrepreneurship support programs, meaning in life, nascent entrepreneurial intention, China, entrepreneurial self-efficacy, opportunity recognition

## Abstract

This study aimed to investigate the impact of entrepreneurial educational support (NEES), entrepreneurial activities support (NEAS), and entrepreneurial commercialization support (NECS) on the nascent entrepreneurial intention (NEI) by mediating roles of entrepreneurial self-efficacy (NESE), opportunity recognition (OR), and the moderating effect of meaning in life (MLI). Data were gathered using a survey questionnaire from the 868 management, engineering, technical, and vocational institute students of China. The NEI model was analyzed using the partial least squares structural equation modeling through Smart-PLS software. The findings of the study reveal that NEES, NEAS, and NECS have a positive effect on NEI. Meanwhile, results indicate that NESE and OR partially mediate the relationship between entrepreneurship support programs and nascent entrepreneurial intention. Furthermore, the relationship between NESE and the NEI was insignificantly influenced by MLI, and the relationship between OR and the NEI was significantly moderated by MLI. Lastly, implications and limitations are also discussed in this article.

## Introduction

The role of entrepreneurship support programs is considered as one of the main factors in creating optimistic expectations of capabilities for new business start-up ventures (Scott et al., 2016), cultivating desirable self-employment behaviors (Krueger and Brazeal, 1994), and ambitions for entrepreneurship (Chen et al., 1998; Nambisan et al., 2018). Given the growing interest from students in academic entrepreneurship and new venture creation, relatively less systematic work has established entrepreneurship education and the support factors that can promote entrepreneurship among university students (Rauch and Hulsink, 2015; Wu et al., 2016; Chen and Zhou, 2017).

Prior research attempted to investigate the effectiveness of entrepreneurial education in the context of Saudi universities, and results were found to be satisfactory, including student satisfaction and success in the entrepreneurship course, which may be inadequate constructs of entrepreneurial educational effectiveness (Ge et al., 2017; Ayed, 2020). Moreover, Fellnhofer and Puumalainen (2017) showed that involvement in an entrepreneurship program substantially increased the perceived viability of starting up a company. Entrepreneurial education teaches the various aspects of starting a new business through a series of courses and practical activities that focus on providing knowledge and practical skills to increase the likelihood of entrepreneurial success (Tarling et al., 2016). The necessity and importance of entrepreneurial education are well emphasized in the existing literature (Liu et al., 2019; Gianiodis and Meek, 2020). It is believed that entrepreneurs can be nurtured through entrepreneurship education (Ronstadt, 1985; Garavan and O’Cinneide, 1994; Gibb, 2002). Also, it is necessary to carry out entrepreneurship education because it can support and formulate the entrepreneurial intention (Entrialgo and Iglesias, 2016). Furthermore, entrepreneurial education reduces the chances of start-up failures and well-informed the entrepreneur with market information asymmetries (McGrath, 1999).

Existing studies have argued that entrepreneurial support programs are essential because they provide an individual to experience exploration and revitalization (Parker, 2018), and provide an individual with opportunities to improve their confidence in entrepreneurship (Karimi et al., 2016). However, despite the necessity and positive recognition of entrepreneurial education, there is still a lack of consistent evidence on entrepreneurial education that can help to foster more or better entrepreneurs (Nowiński et al., 2019). Entrepreneurial education and an entrepreneurial cognitive mindset have a positive relationship (Costa et al., 2018). Thus, it can change the negative perception of entrepreneurship with the medium of education and awareness programs. It replaces the uncertainty of the entrepreneur with knowledge through systematic education programs (Nabi et al., 2017). The education support program is beneficial because it helps to induce entrepreneurial intention and spirit (Kierulff, 2005; Michaelides and Benus, 2012).

Entrepreneurship support programs enhance the entrepreneurial knowledge of individuals, reducing their perceived uncertainty and improving their confidence (Mozahem and Adlouni, 2021). Numerous previous studies have discussed the cognitive factors of entrepreneurial intention, including demographic attributes (Sanoubar et al., 2018), parent and grandparent background (Li and Liu, 2019), role models (Leiter, 1993), entrepreneurial self-efficacy (NESE) (Tsai et al., 2016), locus of control (Leonelli et al., 2016), self-confidence (Willness and Bruni-Bossio, 2017), need for achievement (Dy et al., 2017), market experience (Anwar and Daniel, 2016), and personality characteristics (Travis and Freeman, 2017). The research on entrepreneurship support programs is empirically less examined by the researchers in the literature. Therefore, this study provides some contributions to prior literature on entrepreneurship. First, a few studies have provided evidence that entrepreneurship support programs and NESE can enhance self-confidence or inspire positive emotion (Hamzah et al., 2016; Nabi et al., 2018; García-Vidal et al., 2019). However, there is a lack of an integrated perspective when exploring the intervening mechanism in the relationship between entrepreneurship education programs, NESE, opportunity recognition (OR), and entrepreneurial intention.

Second, particularly in entrepreneurship literature, entrepreneurship education programs, such as entrepreneurship education support, entrepreneurship activities support, and entrepreneurship commercialization support on NESE, OR, and entrepreneurial intention, are regarded as central emotion and cognition fueling entrepreneurial outcomes. Third, the research on entrepreneurship support programs and entrepreneurial intention is vague (Kraaijenbrink et al., 2010). Most of the prior studies examined the direct effect of entrepreneurship education on entrepreneurial intention (Turner and Gianiodis, 2018; Bazkiaei et al., 2020), NESE (Nowiński et al., 2019), and entrepreneurial intention/behavior (Elliott et al., 2020; Wardana et al., 2020). Fourth, the mediating role of NESE and OR in the relationship between entrepreneurship support programs and nascent entrepreneurial intention (NEI) is still under-exported. Fifth, this study introduces the moderating effect of meaning in life (MLI) in the relationship between NESE, OR, and NEI. This relationship is not tested by prior researchers in the literature. Therefore, to fill this research gap, this study contributes to the existing literature on entrepreneurship support programs on NEI among university students.

## Theory and hypotheses development

Entrepreneurial intention defines the link between both ideas and actions that are essential to analyze the process of entrepreneurship (Bird, 1988; Krueger and Carsrud, 1993). According to Ajzen (1991), the intention shows how motivated and willing people carry out the desired behavior. The intention has also been defined as a mindset that focuses on personal attention to achieve something toward a particular goal or path. The intention is also proved as the main determinant of planned behavior (Bagozzi et al., 1989), specifically when it is unique, difficult to track, or has unexpected time delays (Krueger and Carsrud, 1993). A new business has arisen over time with extensive preparation, and thus, entrepreneurship is precisely the form of expected behavior. The position to the action reflects the personal desirability of a new business being evaluated by individuals. In contrast, subjective norms reveal the perception of individuals regarding what is the opinion of people about the importance of entrepreneurial development in their lives. Perceived behavioral control demonstrates the perception that individuals can successfully start a new business.

### Entrepreneurship support programs and entrepreneurial intention

Universities are playing an important role in enhancing the entrepreneurship of university students and implementing entrepreneurship through various entrepreneurship support programs, such as entrepreneurial education, special lectures, awareness session, club activities for concept development, and commercialization support (Maresch et al., 2016; Minai et al., 2018). Entrepreneurial education has a significant impact on developing an entrepreneurial attitude toward entrepreneurial intention to start a new business (Schwarz et al., 2009). In addition to entrepreneurial education, entrepreneurship support programs, such as entrepreneurial activity support and entrepreneurial commercialization support (NECS), raise the possibility of actual nascent entrepreneurial ventures for university students. Therefore, there is no doubt that the entrepreneurship support program offered by the university is very effective in increasing the capacity of university students to start their businesses (Krueger and Brazeal, 1994; Fuller et al., 2018).

Based on the prior literature, it is suggested that entrepreneurship support programs have a positive influence on entrepreneurial intention and venture creation (Gerbin and Drnovsek, 2016; Tran and Von Korflesch, 2016). An existing study by Maresch et al. (2016) argued that in the case of university students, no matter how high the entrepreneurial intention to start a business, it is difficult to actively start a business while simultaneously studying. Therefore, it is difficult for university students to activate their nascent entrepreneurial ventures if they do not create a supportive environment for start-ups or actively support their start-up-related activities (Moser et al., 2017). Primarily, university students’ attitude toward start-ups is not as strong as they think (Bergmann et al., 2016), and the idea of immediate interests is ahead of business continuity. Also, there are various disadvantages and inconveniences in utilizing various kinds of entrepreneurship support programs (Cho et al., 2016). Nevertheless, if universities can provide college students with a strong entrepreneurial intention to start their businesses and provide them with everything they need, it will be easier for university students to become an entrepreneur (Bergmann et al., 2016). Therefore, this study established the following hypothesis.

H1a: Entrepreneurial educational support (NEES) has a positive and significant impact on NEI.

H1b: Entrepreneurial activities support (NEAS) has a positive and significant influence on NEI.

H1c: Entrepreneurial commercialization support has a positive and significant influence on NEI.

### Entrepreneurship support programs and entrepreneurial self-efficacy

Entrepreneurial university growth is a common trend worldwide, which has drawn policymakers’ consideration. Entrepreneurial universities are respected for their economic contributions (such as trademarks, licenses, and start-up businesses) and processes of knowledge transfer (Etzkowitz and Zhou, 2017). A large amount of scholarship has shown universities as seedbeds for cultivating an entrepreneurial spirit and environment. Universities play a significant role in recognizing and improving the entrepreneurial qualities of students and enabling them to start their businesses, thus contributing effectively to economic growth and job development (Henry et al., 2017). Universities, therefore, have an important role in developing new entrepreneurial ventures by promoting a competitive climate and making a major contribution to the economy and society.

Moreover, existing literature has acknowledged the entrepreneur’s educational importance and assistance in creating beneficial views of the competence of start-up firms (Scott et al., 2016). Entrepreneurial education has been correlated with improved behaviors and new start-up plans (Krueger and Brazeal, 1994; Hartshorn and Hannon, 2005). Indeed, university students who took classes in entrepreneurship were more involved in being entrepreneurs relative to those who did not (Kolvereid and Moen, 1997; Neto et al., 2017). University support policies and activities may help the start-ups of students, such as technology transfer offices, business incubators, physical capital, and university venture fund. A successful entrepreneurial education curriculum and university entrepreneurial encouragement are powerful ways to gain the requisite information regarding entrepreneurship and inspire young people to pursue an entrepreneurial future (Van der Zwan et al., 2016).

Furthermore, Liguori et al. (2018) found that many students’ entrepreneurial aspirations are thwarted by inadequate preparation, their market experience is insufficient, and more significantly, they are not prepared to take chances to realize their dreams. Shamsudeen et al. (2017) proposed that entrepreneurial education is successful because it helps participants to build a higher creativity capacity and the desire to think conceptually and see the transition as an opportunity. Kraaijenbrink et al. (2010) indicated that it would be necessary to quantify the magnitude of their effect on students, even though universities would promote entrepreneurship in other objectively calculated ways, to recognize the influence of such initiatives. There are different types of entrepreneurship support programs. First, universities should provide the general knowledge and skills required to implement a new project to display entrepreneurial skills as part of their typical teaching function. Second, considering their marketing position, universities may offer more precise and focused resources to individual students or groups of students to start their entrepreneurial ventures (Lim et al., 2016; Mustafa et al., 2016). Krueger and Brazeal (1994), proposed that entrepreneurial education would boost perceived entrepreneurship feasibility by growing learner awareness and confidence and promoting self-efficacy. This implies the enterprise services and related resources offered by universities will play a significant role in fostering NESE among their students.

H2a: Entrepreneurial educational support has a positive impact on entrepreneurial self-efficacy.

H2b: Entrepreneurial activities support has a positive influence on entrepreneurial self-efficacy.

H2c: Entrepreneurial commercialization support has a positive effect on entrepreneurial self-efficacy.

### Entrepreneurship support programs and opportunity recognition

A few empirical studies have examined the relationship between entrepreneurship support programs and awareness of OR. Ardichvili et al. (2003) asserted the integrated model of recognition of entrepreneurial opportunities and entrepreneurial intention. The identification and creation of opportunities that led to the establishment of enterprises include entrepreneurial alertness, information asymmetry and prior knowledge, social networks, personality traits, self-efficacy, creativity, and the opportunity itself. The mechanism of this relationship leads to a certain degree of entrepreneurial alertness and the creation of entrepreneurial opportunity, which involves recognition and evaluation. Moreover, Wright et al. (2017) found a direct relationship between entrepreneurial education and recognition of entrepreneurial opportunities. Li et al. (2020) argued that individuals with a high-level capacity to recognize the opportunities are more likely to exploit those opportunities in the market. OR involves four activities: recording the opportunities encountered during the day; creating opportunities through their introduction, teamwork, and the like; disclosing and contemplating one’s ideas through brainstorming and the like to achieve crucial conception; and creating inclination to new challenges through the experience of failure. Thus, based on the discussion, this study hypothesized;

H3a: Entrepreneurial educational support has a positive effect on opportunity recognition.

H3b: Entrepreneurial activity has a positive impact on opportunity recognition.

H3c: Entrepreneurial commercialization has a positive impact on opportunity recognition.

### Entrepreneurial self-efficacy and entrepreneurial intention

Prior studies argued that NESE provides a significant impact on entrepreneurial intention (Naktiyok et al., 2010; Hsu et al., 2019; Xiaoping et al., 2019). NESE refers to an individual’s belief in his/her capability to perform tasks and roles aimed at entrepreneurial outcomes (Li et al., 2020; Jiatong et al., 2021a). Several studies examined the role of NESE on the university entrepreneurial intention of students (Rosique-Blasco et al., 2018; Asimakopoulos et al., 2019). Universities do not reflect the consideration of a relationship between entrepreneurial intention and actual start-ups when developing entrepreneurship support programs. Universities’ entrepreneurship support programs are developed during their respective semester, as programs offered after graduation are not considered to be undertaken by students (Galloway and Brown, 2002; Debarliev et al., 2020). If an individual intends to start their businesses, they can raise their intentions by taking classes, training, or joining an entrepreneurial club (Peterman and Kennedy, 2003; Nowiński et al., 2019). The students who experienced various entrepreneurship support programs implemented by the university showed higher entrepreneurial intentions (Souitaris et al., 2007).

Moreover, university students can reinforce their intention to start a new business by directly or indirectly experiencing cases related to the start-up of their parents or acquaintances. In other words, when university students experience the role model related to entrepreneurship, their intention to start up can be increased (Sequeira et al., 2007; Elliott et al., 2020). Therefore, individuals with a higher level of NESE are more likely to start a new business venture (Krueger, 1993; Yang, 2019). Therefore, this study established the following hypothesis.

H4: Entrepreneurial self-efficacy has a positive effect on entrepreneurial intention.

### Opportunity recognition and entrepreneurial intention

The role of OR in entrepreneurship has been recognized by prior researchers (Alvarez and Barney, 2007; Mary George et al., 2016). Entrepreneurship is not only found through opportunity identification but also through OR and discovery (Shane, 2003). OR is an objective opportunity to be found by creative people from the perspective of Schumpeter’s innovation (Drejer, 2004). On the other hand, OR has been created through the ideas, interactions, or exchanges among people from the perspective of the individuals. It is recognized that the OR obtained through the entrepreneurship support program leads to entrepreneurial intention. Therefore, students who received entrepreneurial education were assessed on different levels for OR (Ramos-Rodriguez et al., 2010; Asante and Affum-Osei, 2019). Therefore, this study argued that entrepreneurship support programs help students to improve their ability to identify and exploit the entrepreneurial opportunity to become entrepreneurs (Kang et al., 2016). Therefore, this study established the following hypothesis.

H5: Opportunity recognition has a positive influence on entrepreneurial intention.

### Mediating effect of entrepreneurial self-efficacy and opportunity recognition

The relationship between NESE and OR has been studied by prior researchers (Tumasjan and Braun, 2012; Urban and Galawe, 2020). NESE and OR are described as the individual’s belief and ability to identify the new and productive ideas from the entrepreneurial intention (Al-Shammari and Al Shammari, 2018). NESE and OR are the central practices of entrepreneurship that provide knowledge to an individual for identifying the business needs and constantly analyzing relevant skills gained through creative ideas (Tsai et al., 2016; Guo et al., 2017). Entrepreneurs gain skills through entrepreneurship training, recognize successful expertise from a vast volume of content, transform input into new goods or services, develop new markets, maximize growth potential, and lead to team building (Mustafa et al., 2016). Therefore, an individual with a high level of NESE is more likely to cope with and control the challenges and risks that can arise during the start-up process (Krueger and Brazeal, 1994; Solesvik, 2017). Thus, we believed that self-efficacy and OR are useful predictors for measuring entrepreneurial intention, because individuals with a higher level of NESE identify the entrepreneurial opportunity, to start a new business venture (Jiang et al., 2017). Therefore, the intention to start a business is likely to vary depending on NESE and OR (Brändle et al., 2018). Consequently, this study formulated the following hypotheses.

H6a: Entrepreneurial self-efficacy positively mediates the relationship between entrepreneurial educational support and entrepreneurial intention.

H6b: Entrepreneurial self-efficacy positively mediates the relationship between entrepreneurial activity support and entrepreneurial intention.

H6c: Entrepreneurial self-efficacy positively mediates the relationship between entrepreneurial commercialization support and entrepreneurial intention.

H7a: Opportunity recognition mediates the relationship between entrepreneurial educational support and entrepreneurial intention.

H7b: Opportunity recognition mediates the relationship between entrepreneurial activity support and entrepreneurial intention.

H7c: Opportunity recognition mediates the relationship between entrepreneurial commercialization support and entrepreneurial intention.

### The meaning in life and entrepreneurial intention

There is a great saying by William James, “Do not be scared of creation. Believe that life is worth living and the reality would be produced by your conviction” (James, 1956). MLI, in the true sense, is a double-edged sword. Such a common conviction is practical in certain cases and enhances the sense of optimism and intent of the nation. Nevertheless, in stressful circumstances, these individuals suffer from broken expectations when this conviction is undermined by the grim truth (Brandt and Proulx, 2016). According to MLI implies a deep conviction that is a certain form of action or the condition of being personal or socially more acceptable than the alternative type of conduct. The relationship between MLI and entrepreneurial intention is less empirically examined by researchers in the field of entrepreneurship. Therefore, it is not impossible to believe that entrepreneurship ability affects human lives. MLI may play an invigorating function in the transition from idea to execution. Thus, we believed that MLI might foster entrepreneurial intention. So, we predicted the following hypothesis.

H8: The meaning in life has a positive impact on entrepreneurial intention.

### Moderating effect of meaning in life

Meaning in life is not meant to be delivered explicitly, but instead an interaction that encompasses something special. To be able to frame this something else in terms of life means some sort of representation has to be made. According to Frankl (1985), MLI is the underlying motivation for human behavior, which helps one to attain spiritual wellness. Based on the taxonomy of self-sense experiences, it was proposed that value in existence should be strongly associated with assumptions regarding entrepreneurial intention. Under other terms, people with greater MLI will have better support for entrepreneurial self-efficacy, OR, and entrepreneurial intention. In specific, MLI was hypothesized to strengthen entrepreneurial self-efficacy and OR, which ultimately would affect entrepreneurial intention.

H9: The meaning in life moderates the relationship between entrepreneurial self-efficacy and entrepreneurial intention.

H10: The meaning in life moderates the relationship between opportunity recognition and entrepreneurial intention.

#### Conceptual model

**FIGURE 1:**
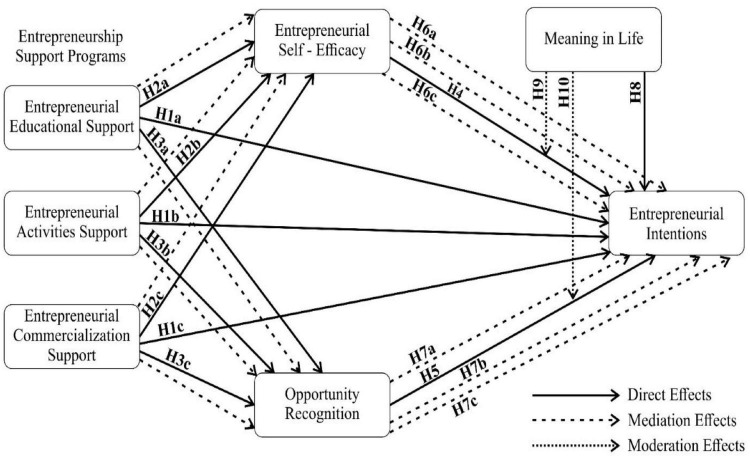
Conceptual model.

The conceptual model depicting the relationships and hypothesis is given in Figure 1.

## Materials and methods

### Sample and data collection

Considering the relevance of education, the current study attempted to investigate the effect of entrepreneurship support programs on formulating entrepreneurial intentions in the higher education sector of China. Higher education in this context refers to universities and technical and vocational institutions. A purposive sampling technique was used because this technique enables researchers to squeeze a lot of information out of the data that they have collected from the sample. This study targeted the management, engineering, technical, and vocational institute students of China. We did not select faculty members for data collection because they do not have much experience to implement changes to support entrepreneurship programs. Moreover, students were the main respondents, as they are better aware of entrepreneurship support program scenarios in their respective environmental settings. We randomly distributed the questionnaire among the students during their free time to enhance the quality of responses. The survey was conducted from 1 January 2020 to 20 February 2020. The data were collected using time lags of 5 weeks between the two rounds. In the first 2 weeks, we collected data for the entrepreneurship support programs. For the remaining 2 weeks, the same procedure was adopted from the same respondents for the data collection of other predictors, such as NESE, OR, MLI, and entrepreneurial intention to minimize the issue of common method bias. Among the valid questionnaires, 450 (51.8%) were filled by men and 418 (48.1%) by women. The mean age was 1.78 years with a standard deviation of 0.829. The education of students falls between different disciplines: bachelor’s (46.1%), master’s (19.4%), Ph.D. (2.3), and vocational diploma (32.0%).

### Measures

This study used measurement scales that had been tested and validated by previous researchers. A questionnaire comprising a total of 35 items was utilized to estimate the effect of each scale. All the items were ranked on a seven-point Likert scale ranging from strongly disagree (1) to strongly agree (7).

#### Nascent entrepreneurial intention

To assess NEI, we used five items from a prior study (Li et al., 2020). This scale was used by existing researchers to examine the entrepreneurial intention of students (Liñán and Chen, 2009; Jiatong et al., 2021b). A sample item is “I am ready to do anything to be an entrepreneur.”

#### Entrepreneurship support programs

To measure the entrepreneurship support programs of university students, the entrepreneurship support programs scale was divided into three dimensions: entrepreneurial educational support, NEAS, and NECS.

##### Entrepreneurial educational support

To measure NEES, we used five items from a previous study (Omer and Aljaaidi, 2020). A sample item is “The University offers elective courses on entrepreneurship.”

##### Entrepreneurial activities support

To evaluate entrepreneurial activities support, we used four measurement items from a previous study (Omer and Aljaaidi, 2020). A sample item is “My University sipper the entrepreneurial activities and encouraged me to consider taking risks.”

##### Entrepreneurial commercialization support

To assess NECS, we adopted five items from a previous study (Jiatong et al., 2021b). A sample item is “My University focuses on the commercialization of innovative ideas and promotes entrepreneurship.”

#### Entrepreneurial self-efficacy

To measure NESE, three items were used on a seven-point Likert scale. The items were adopted from a previous study (Zhao et al., 2005). A sample item is “I am convinced that I can successfully create new products.”

#### Opportunity recognition

To assess OR, we used five-item scales from the study by Kuckertz et al. (2017). A sample item is “I am always alert to business opportunities.”

#### Meaning in life

Meaning in life was measured based on Frankl’s theory, and all the items were measured on a seven-point Likert scale. The measurement scale for MLI was adopted from the study by Steger et al. (2006). A sample item is “I am seeking a purpose or mission for my life.”

### Measurement model

The measurement model analysis was performed using Cronbach’s alpha, composite reliability, and average variance extracted. Table 1 shows that all the values of Cronbach’s alpha, composite reliability, and average variance extracted were satisfactory and according to the suggested benchmark > 0.70 and >0.50 by previous researchers (Bagozzi and Yi, 1988; Hair et al., 2012; Fuller et al., 2018; Cai et al., 2021).

**TABLE 1:** Measurement model.

Variables and constructs	Loadings	Cronbach’s alpha	Composite reliability	AVE
NEI		0.946	0.959	0.824
NEI 1	0.930			
NEI 2	0.918			
NEI 3	0.924			
NEI 4	0.989			
NEI 5	0.866			
NEES		0.949	0.961	0.832
NEES 1	0.878			
NEES 2	0.899			
NEES 3	0.942			
NEES 4	0.923			
NEES 5	0.916			
NEAS		0.941	0.958	0.850
NEAS 1	0.915			
NEAS 2	0.941			
NEAS 3	0.918			
NEAS 4	0.913			
NECS		0.959	0.976	0.891
NECS 1	0.959			
NECS 2	0.930			
NECS 3	0.953			
NECS 4	0.950			
NECS 5	0.926			
NESE		0.900	0.938	0.834
NESE 1	0.938			
NESE 2	0.918			
NESE 3	0.881			
OR		0.957	0.967	0.854
OR 1	0.918			
OR 2	0.920			
OR 3	0.921			
OR 4	0.936			
OR 5	0.925			
MLI		0.971	0.978	0.897
MLI1	0.956			
MLI 2	0.949			
MLI 3	0.965			
MLI 4	0.930			
MLI 5	0.935			

NEES, entrepreneurial educational support; NEAS, entrepreneurial activities support; NECS, entrepreneurial commercialization support; NESE, entrepreneurial self-efficacy; OR, opportunity recognition; MLI, meaning in life; NEI, nascent entrepreneurial intention.

#### Validity test

To test the construct validity, this study used two criteria: Fornell–Larcker and heterotrait-monotrait ratio (HTMT). Both criteria were widely used for assessing discriminant validity (Henseler et al., 2015; Shirokova et al., 2016). The results presented in Tables 2, 3 indicate that the values of Fornell–Larcker and HTMT were acceptable.

**TABLE 2:** Fornell–Larcker criteria.

	MLI	MLI*NESE and NEEI	MLI*OR and NEEI	NEAS	NECS	NEEI	NEES	NESE	OR
MLI	0.947	–		–	–				
MLI*NESE and NEEI	–0.318	1.000		–	–				
MLI*OR and NEEI	–0.305	0.333	1.000						
NEAS	0.426	–0.357	–0.171	0.922	–				
NECS	0.327	–0.057	–0.106	0.378	0.944				
NEEI	0.379	–0.214	–0.081	0.390	0.404	0.908			
NEES	0.410	–0.321	–0.142	0.519	0.301	0.393	0.912		
NESE	0.400	–0.353	–0.145	0.508	0.328	0.385	0.515	0.913	
OR	0.440	–0.151	–0.315	0.363	0.475	0.475	0.352	0.345	0.924

NEES, entrepreneurial educational support; NEAS, entrepreneurial activities support; NECS, entrepreneurial commercialization support; NESE, entrepreneurial self-efficacy; OR, opportunity recognition; MLI, meaning in life; NEI, nascent entrepreneurial intention.

**TABLE 3:** Heterotrait-monotrait ratio criteria.

	MLI	MLI*NESE and NEEI	MLI*OR and NEEI	NEAS	NECS	NEEI	NEES	NESE	OR
MLI		–		–	–				
MLI*NESE and NEEI	0.322			–	–				
MLI*OR and NEEI	0.310	0.333							
NEAS	0.445	0.367	0.177		–				
NECS	0.336	0.059	0.108	0.395					
NEEI	0.394	0.210	0.083	0.411	0.420				
NEES	0.427	0.329	0.146	0.548	0.314	0.413			
NESE	0.428	0.373	0.153	0.550	0.349	0.415	0.557		
OR	0.456	0.154	0.322	0.381	0.492	0.452	0.370	0.369	–

NEES, entrepreneurial educational support; NEAS, entrepreneurial activities support; NECS, entrepreneurial commercialization support; NESE, entrepreneurial self-efficacy; OR, opportunity recognition; MLI, meaning in life; NEI, nascent entrepreneurial intention.

## Results

### Direct effects

**FIGURE 2:**
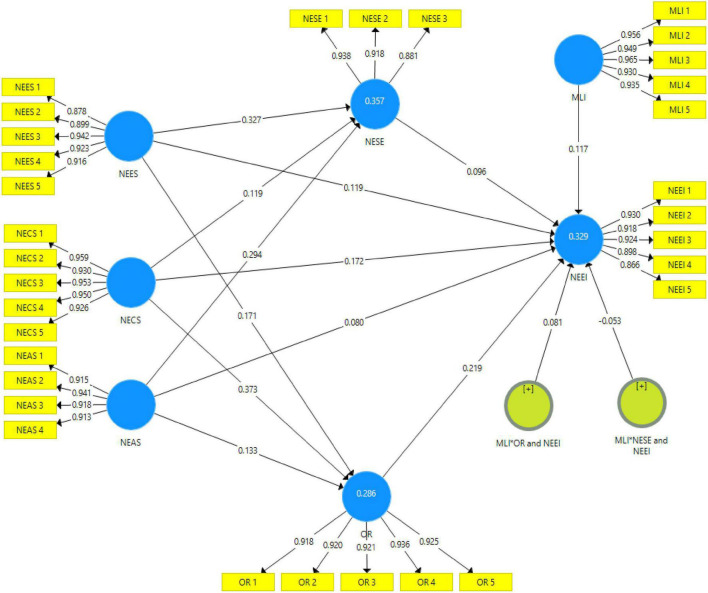
Structural model.

The results were analyzed through Smart-PLS 3.3.9 software, and the partial least squares structural equation modeling technique was adopted for the estimation of the structural model. The SEM technique incorporates measurement error and provides best-suited predictions of interaction effects, such as direct, mediation, and moderation (Henseler et al., 2015; Li et al., 2020). The results of the hypotheses are presented in Table 4 and Figure 2. The validity testing of H1a shows that NEES had a positive and significant impact on the NEI with a path coefficient beta 0.119, *t*-value 2.398, and *p* < 0.05. Thus, H1a was accepted. Moreover, H1b findings indicate that NEAS had a positive but insignificant effect on the NEI with a path coefficient beta 0.08, *t*-value 1.523, and *p* > 0.010. Therefore, H1b was not supported. In addition, H1c results show that NECS had a positive impact on the NEI with a path coefficient beta of 0.172, *t*-value of 3.536, and *p* < 0.05. Thus, H1c was accepted. Additionally, to test H2a, the empirical findings indicate that NEES had a positive effect on NESE with a path coefficient beta of 0.327, *t*-value of 7.322, and *p* < 0.05. So, H2a was supported. Moreover, H2b results show that NEAS had a positive influence on NESE with a path coefficient beta of 0.294, *t*-value of 7.099, *p* < 0.05. Consequently, H2b was accepted. Meanwhile, H2c results illustrate that NECS had a positive impact on NESE with a path coefficient beta of 0.119, *t*-value of 3.049, and *p* < 0.05. Therefore, H2c was accepted.

**TABLE 4:** Path coefficients (direct effects).

Hypotheses	Relationships	β	*t*	*f* ^2^	*P*	Decision
H1a	NEES- NEI	0.119	2.398	0.013	0.017**	Supported
H1b	NEAS - NEI	0.080	1.523	0.005	0.128	Not Supported
H1c	NECS- NEI	0.172	3.536	0.031	0.000***	Supported
H2a	NEES-NESE	0.327	7.322	0.119	0.000***	Supported
H2b	NEAS-NESE	0.294	7.099	0.091	0.000***	Supported
H2c	NECS-NESE	0.119	3.049	0.019	0.002**	Supported
H3a	NEES-OR	0.171	4.162	0.029	0.000***	Supported
H3b	NEAS-OR	0.133	2.867	0.017	0.004**	Supported
H3c	NECS-OR	0.373	8.375	0.165	0.000***	Supported
H4	NESE - NEI	0.096	2.272	0.008	0.024**	Supported
H5	OR -NEI	0.219	4.635	0.045	0.000***	Supported
H8	MLI - NEI	0.117	2.073	0.013	0.039**	Supported
H9	MLI*NESE -NEI	–0.053	1.621	0.005	0.106	Not Supported
H10	MLI*OR -NEI	0.081	2.424	0.014	0.016**	Supported

***p < 0.01, **p < 0.05, *p < 0.10.

NEES, entrepreneurial educational support; NEAS, entrepreneurial activities support; NECS, entrepreneurial commercialization support; NESE, entrepreneurial self-efficacy; OR, opportunity recognition; MLI, meaning in life; NEI, nascent entrepreneurial intention.

Apart from this, H3a findings show that NEES had a positive impact on OR with a path coefficient beta of 0.096, *t*-value of 2.272, and *p* < 0.05. Thus, H3a was accepted. Moreover, H3b results indicate that NEAS had a positive influence on OR with a path coefficient beta 0.219, *t*-value 4.635, and *p* < 0.05. So, H3b was supported. Meanwhile, H3c results explain that NECS had a positive impact on OR with a path coefficient beta of 0.373, *t*-value of 8.375, and *p* < 0.05. Hence, H3c was supported. Furthermore, H4 results show that NESE had a positive effect on the NEI with a path coefficient beta of 0.096, *t*-value of 2.272, and *p* < 0.05. Therefore, H3c was accepted. Additionally, H5 results show that OR had a positive impact on the NEI with a path coefficient beta of 0.219, *t*-value of 4.635, and *p* < 0.05. Consequently, H5 was accepted.

### Indirect effects (mediation analysis)

Table 5 illustrates the specific indirect effects of the mediation analysis. H6a results show that NESE positively mediates the relationship between NEES and NEI with a path coefficient beta of 0.031, *t*-value of 2.139, and *p* < 0.05. So, H6a was supported. Moreover, H6b findings reveal that NESE partially mediates the relationship between entrepreneurial activity support and NEI with a path coefficient beta of 0.028, *t*-value of 2.202, and *p* < 0.05. Therefore, H6b was accepted. Furthermore, H6c results indicate that NESE positively mediates the relationship between NECS and NEI with a path coefficient beta of 0.011, *t*-value of 2.753, and *p* < 0.05. Hence, H6c was also accepted. Likewise, H7a results explain that OR positively mediates the relationship between NEES and NEI with a path coefficient beta of 0.037, *t*-value of 3.029, and *p* < 0.05. So, H7a was supported. Meanwhile, H7b findings show that OR partially mediates the relationship between entrepreneurial activity support and entrepreneurial intention with a path coefficient beta of 0.029, *t*-value of 2.254, and *p* < 0.05. Thus, H7b was accepted. Lastly, H7c results indicate that OR partially mediates the relationship between NECS and NEI with a path coefficient beta of 0.082, *t*-value of 4.012, and *p* < 0.05. Hence, H7c was also accepted.

**TABLE 5:** Specific indirect effects (mediation analysis).

Hypotheses	Relationships	β	*t*	Confidence interval 2.5%	97.5%	*p*
H6a	NEES- NESE- NEI	0.031	2.139	0.005	0.061	0.033**
H6b	NEAS- NESE- NEI	0.028	2.202	0.004	0.051	0.028**
H6c	NECS- NESE- NEI	0.011	2.735	0.001	0.025	0.050**
H7a	NEES-OR-NEI	0.037	3.029	0.015	0.064	0.003**
H7b	NEAS-OR-NEI	0.029	2.254	0.006	0.060	0.025**
H7c	NECS-OR-NEI	0.082	4.012	0.044	0.125	0.000***

***p < 0.01, **p < 0.05, *p < 0.10.

NEES, entrepreneurial educational support; NEAS, entrepreneurial sctivities support; NECS, entrepreneurial commercialization support; NESE, entrepreneurial self-efficacy; OR, opportunity recognition; MLI, meaning in life; NEI, nascent entrepreneurial intention.

### Moderating effects

The results of the test conducted to determine the moderating effects are presented in Table 4. H8 results indicate that MLI had a positive and significant effect on the NEI with path coefficient beta of 0.117, *t*-value of 2.073, and *p*-value < 0.05. So, H8 was supported. Meanwhile, H9 results show that MLI had a negative and insignificant effect on the relationship between NESE and NEI with a path coefficient beta of –0.053, *t*-value of 1.621, and *p* > 0.010. Therefore, H9 was not supported. Furthermore, H10 findings indicate that MLI had a positive moderating effect on the relationship between OR and NEI with a path coefficient beta of 0.081, *t*-value of 2.424, and *p* < 0.05. Hence, H10 was accepted.

## Discussion

The results of hypotheses H1a, H1b, and H1c indicate that entrepreneurship support programs were positively associated to form NEI s among students. This study finding argued that it is essential to provide entrepreneurship support programs through well-organized entrepreneurial start-up awareness training programs to drive the entrepreneurial spirit among students to do something innovative. Indeed, a prior study explained that students have a lot of innovative ideas, and they require a proper platform to exploit their ideas and be brainstormed, incubated, and transformed into the actual entrepreneurial intention (Nowiński et al., 2019). This result is in line with the prior studies, which found that students with a higher level of entrepreneurial support programs, such as educational support, activities support, and commercialization support, are more likely to engage in forming NEIs (Asimakopoulos et al., 2019; Li et al., 2020). Moreover, it is one of the major issues of developing economies that individuals have limited access to capital and commercialization of their respective innovative ideas in the market. Therefore, universities provide students a platform with techniques and procedures to commercialize their entrepreneurial ideas into reality, which will ultimately result in forming a higher level of NEI s that can later translate into nascent entrepreneurial behavior (Rauch and Hulsink, 2015).

Furthermore, H2a, H2b, and H2c results showed that entrepreneurship support programs significantly influenced NESE. This finding is similar to previous researchers who remarked that entrepreneurship education programs develop entrepreneurial mindset and self-efficacy among individuals to become entrepreneurs (Naktiyok et al., 2010; Liu et al., 2019). NESE acts as a blessing in disguise if there is proper educational support, entrepreneurial activity support, and NECS to develop NEI s.

Additionally, this study found that entrepreneurship support programs are positively related to OR, and its effect is further postulated under hypotheses H3a, H3b, and H3c. This finding is consistent with the existing studies (Ramos-Rodriguez et al., 2010; Ploum et al., 2018). It explains the phenomenon that students perceive entrepreneurship support programs as a trigger to OR that later translates into formulating entrepreneurial intentions. The rise in entrepreneurship support programs facilitates the young minds to identify the available opportunities, and before completing their respective degrees and diplomas, they make up their mind by developing the entrepreneurial intention to kick-start with entrepreneurial ventures.

Besides, H4 results indicated that NESE significantly influenced NEI. This result is consistent with prior studies that argued that students with a higher level of NESE are more engaged to form entrepreneurial intention that ultimately translates into entrepreneurial behavior (Yang et al., 2019; Jiatong et al., 2021b). Meanwhile, H5 findings revealed that OR is positively associated with NEI. This study finding is in line with the existing research (Reid et al., 2018), which remarked that entrepreneurship support programs help individuals to recognize and exploit the entrepreneurial opportunity.

Furthermore, the indirect effects of H6a, H6b, H6c, H7a, H7b, and H7c were also partially mediated in the relationship between entrepreneurship support programs and NESE, OR, and NEI. This finding provides an insightful contribution to the literature on entrepreneurship support programs using NESE and OR as a mediator and found a significant impact on NEI. Lastly, H8, H9, and H10 findings showed that MLI had a positive direct and moderating effect on the relationship between NESE, OR, and NEI. The MLI defines an individual purpose in life, such as whether they are satisfied in their respective lives and what sort of work or future they perceive. This study confirms that individuals who have high MLI are more likely to define the purpose that translates into formulating entrepreneurial intention.

## Theoretical contributions

This study makes the following theoretical implications. First, this study develops the drivers of entrepreneurship support programs, particularly in the context of higher education by identifying the roles of NESE and OR. Prior studies have examined the antecedents of entrepreneurship, such as entrepreneurial traits (Şahin et al., 2019), entrepreneurial alertness (Li et al., 2020), entrepreneurial mindset (Jiatong et al., 2021b), and entrepreneurial education (Elliott et al., 2020). Entrepreneurship support programs in terms of educational support; NEAS, and commercialization support help students in designing the entrepreneurial start-up feasibility, providing financial support in terms of seed funds to promote entrepreneurial ideas that ultimately translate into forming an entrepreneurial intention.

Second, by investigating the mediating roles of NESE and OR, this study contributes a better understanding of the intervening mechanisms under which entrepreneurship support programs influence NEI. Prior research has highlighted the mediating role of entrepreneurial passion (Syed et al., 2020) and entrepreneurial orientation (Ciampi et al., 2021) in the process of entrepreneurial decision-making while ignoring the potential effects of NESE and OR, which are seen as central indicators that drive entrepreneurship. Prior researchers call for the study to identify the effects of entrepreneurial cognition and passion simultaneously on the relationship between environmental factors and entrepreneurial intentions (Neneh, 2020; Chandra et al., 2021).

Third, this study contributes to the literature on entrepreneurship support programs stimulating NESE to develop NEI among management, engineering, technical, and vocational students. Previous studies have examined the effect of entrepreneurial social identity centrality on nascent entrepreneurial behavior (Murnieks et al., 2020; Gur and Mathias, 2021). The results of this study revealed that entrepreneurship support programs have a significant effect on NESE and OR, which answered the call to examine the antecedents of NESE and OR.

Fourth, this study extends the literature on MLI in the relationship between NESE, OR, and NEI. Existing studies have investigated the role of proactive personality and anticipated regret on entrepreneurial intention/behavioral models (Li et al., 2020; Neneh, 2020). Therefore, this study contributes to the literature on entrepreneurship and found a significant moderating impact on MLI among young entrepreneurs who want to start a new business venture.

### Practical implications

The study provides some practical implications for educators, universities, and policymakers. First, universities should implement measures to reinforce entrepreneurship support programs and develop NESE among students, and also focus on enhancing confidence and passion to identify the entrepreneurial opportunity. Universities introduce various entrepreneurship support programs, arrange entrepreneurial competitions, and provide capital support and technical guidance for students to engage in entrepreneurial start-up activities. Second, universities should arrange some entrepreneurial seminars and invite corporate leaders to conduct fruitful lecturers for students. Universities made entrepreneurial education compulsory for every undergraduate and graduate degree to resolve the accelerating issue of unemployment. Third, the government should launch some entrepreneurship support programs to facilitate the entrepreneurs and start-ups with micro-finance loans at easy installments. To stimulate entrepreneurship, the government should establish business incubator centers for students and provide financial support to articulate their business ideas into reality. This reflects the bright chances of generating abundant young minds with higher entrepreneurial intentions. Fourth, universities and colleges should adopt different teaching programs to improve the NESE of different groups of students through categorized teaching and guidance, so that they can find an effective orientation that meets their entrepreneurial intentions. Universities should actively integrate the current entrepreneurship support programs and cases into the on-campus entrepreneurship education, so that the support from the external environment of entrepreneurship can be effectively transformed into the improvement of NESE and OR of college students, which can in turn act positively on their NEI s and behaviors. Lastly, universities and other academic forums design the best entrepreneurship support programs for students, so before the student enters the job market they must have concrete entrepreneurial venture parallel established.

## Limitations and future research directions

There are some limitations and future research directions of our study should be acknowledged. First, in contrast to most of the studies in the prior literature, our study focus is on NEI s rather than actual entrepreneurial behavior. An existing study has revealed a positive relationship between perceived university support and entrepreneurial intention using the theory of planned behavior as a mediator (Liu et al., 2022). However, there is still a gap between other entrepreneurship support programs and actual entrepreneurial behavior. Our study is unable to investigate how many students will eventually transform their NEI s into entrepreneurial behavior to start a new business. Therefore, in the future, a cross-sectional and longitudinal study could reveal a better understanding of whether entrepreneurial intentions could turn into a behavior. Second, the generalizability of the current findings may be limited. As is often the case, this study was conducted within one country. Our framework provides a meaningful understanding of the topic, and future research could survey a more diverse sample that comes from different cultures and countries. Third, since cultural disparities might be responsible for the non-significant relationship between attitudes toward entrepreneurship and entrepreneurial intentions, future studies could also investigate whether there are potential specific motives and advantages for university students to start a new business in China, taking government policy and culture context into consideration. Future research might reflect on the other entrepreneurial aspects, e.g., the role of business incubators and entrepreneurship development context using NESE as a mediator. In addition, further research needs to identify the role of social networks as a mediating factor to explore the influence of entrepreneurship orientation on NEI.

## Data availability statement

The raw data supporting the conclusions of this article will be made available by the authors, without undue reservation.

## Ethics statement

The studies involving human participants were reviewed and approved by Zhaoqing University, Zhaoqing, Guangdong, China. The patients/participants provided their written informed consent to participate in this study.

## Author contributions

SL and HJ proposed the research idea, analyzed the results, and wrote the manuscript. LS and MA carried out the methodology and extensively edited the manuscript. All authors contributed to the article and approved the submitted version.

## Conflict of interest

The authors declare that the research was conducted in the absence of any commercial or financial relationships that could be construed as a potential conflict of interest. The handling editor declared a past co-authorship with one of the author, HJ.

## Publisher’s note

All claims expressed in this article are solely those of the authors and do not necessarily represent those of their affiliated organizations, or those of the publisher, the editors and the reviewers. Any product that may be evaluated in this article, or claim that may be made by its manufacturer, is not guaranteed or endorsed by the publisher.
